# Identification of a 5-Gene Metabolic Signature for Predicting Prognosis Based on an Integrated Analysis of Tumor Microenvironment in Lung Adenocarcinoma

**DOI:** 10.1155/2020/5310793

**Published:** 2020-06-26

**Authors:** Xiaolin Yu, Xiaomei Zhang, Yanxia Zhang

**Affiliations:** ^1^Beijing University of Chinese Medicine, Beijing 100029, China; ^2^Department of Respiratory, Dongfang Hospital, Beijing University of Chinese Medicine, Beijing 100078, China

## Abstract

Lung adenocarcinoma (LUAD) is a common subtype of lung cancer with a depressing survival rate. The reprogramming of tumor metabolism was identified as a new hallmark of cancer in tumor microenvironment (TME), and we made a comprehensive exploration to reveal the prognostic role of the metabolic-related genes. Transcriptome profiling data of LUAD were, respectively, downloaded from the Cancer Genome Atlas (TCGA) and Gene Expression Omnibus (GEO) database. Based on the extracted metabolic-related genes, a novel 5-gene metabolic prognostic signature (including GNPNAT1, LPGAT1, TYMS, LDHA, and PTGES) was constructed by univariate Cox regression and least absolute shrinkage and selection operator (LASSO) regression. This signature confirmed its robustness and accuracy by external validation in multiple databases. It could be an independent risk factor for LUAD, and the nomograms possessed moderately accurate performance with the C-index of 0.755 (95% confidence interval: 0.706–0.804) and 0.691 (95% confidence interval: 0.636–0.746) in training set and testing set. This signature could reveal the metabolic features according to the results of gene set enrichment analysis (GSEA) and meanwhile monitor the status of TME through ESTIMATE scores and the infiltration levels of immune cells. In conclusion, this gene signature is a cost-effective tool which could indicate the status of TME to provide more clues in the exploration of new diagnostic and therapeutic strategy.

## 1. Introduction

Lung cancer has become one of the most frequently diagnosed malignant tumors with a leading death rate [1]. The major histological subtype of lung cancer is non-small-cell lung cancer (NSCLC) accounting for approximately 85% [2, 3]. Lung adenocarcinoma (LUAD) was the most common subtype of NSCLC with the 5-year survival rate of about 15% [4, 5]. The studies on the driver oncogenes such as epidermal growth factor receptor (EGFR) and anaplastic lymphoma kinase (ALK) have got great achievements [6]. However, the drug resistance of targeted therapy against these genes was usually the inevitable limitation to patients, and novel mechanisms of therapy were urgent to be explored for clinical practice [7].

The reprogramming of tumor metabolism was identified as a new hallmark of cancer in tumor microenvironment (TME) [8]. In the background of TME, the disorder of tumor metabolism could deeply influence the multiple functions of malignant cancer cells [9]. Previous reports have identified metabolic signatures for prognostic prediction based on multiomics analyses in lung cancer [10–12]. However, the TME is a complex interaction network, and the integrated research on the roles of metabolic signatures in TME is still lacking.

In the current research, we constructed a novel metabolic-related gene signature to reflect the status of TME. Based on the differentially expressed metabolic genes of the TCGA cohort, we confirmed the potential prognostic values of this signature. It could reflect the metabolic features of LUAD and further monitor the content of stromal and immune cells. We aimed to provide new clues and directions for further research on the genes that participated in TME.

## 2. Materials and Methods

### 2.1. Data Collection

The normalized mRNA transcriptome profiling data of LUAD were downloaded from the Cancer Genome Atlas (TCGA) database (https://portal.gdc.cancer.gov/) and GSE72094 dataset from the Gene Expression Omnibus (GEO) database (https://www.ncbi.nlm.nih.gov/geo/) [13]. The TCGA cohort contained 535 LUAD samples and 59 control samples and the GSE72094 cohort contained 442 LUAD samples. The corresponding clinical features were also obtained and extracted. Genes that were involved in metabolism pathways were selected as metabolic genes according to the Kyoto Encyclopedia of Genes and Genomes (KEGG) pathway gene sets downloaded from MSigDB database (http://software.broadinstitute.org/gsea/msigdb). The intersection of genes among these three datasets was prepared as metabolic-related genes for subsequent analyses.

### 2.2. Identification of Differentially Expressed Metabolic Genes

First, the mRNA expression matrices of the TCGA and GEO cohorts were normalized, respectively. Then, the differentially expressed metabolic genes in the TCGA cohort were selected by the threshold of |log 2[fold change(FC)]| ≥1 and false discovery rate (FDR) <0.05 via limma package [14].

### 2.3. Construction and Assessment of the Metabolic Gene Signature

The survival-related metabolic genes were extracted by univariate Cox regression analysis with the threshold of *p* < 0.001 by survival package [15]. Samples whose survival time was less than 30 days and with incomplete clinical information were excluded from this analysis. Then, a least absolute shrinkage and selection operator (LASSO) regression was performed to construct the prognostic signature and avoid overfitting of this model by glmnet package [16]. The metabolic gene signature was constructed based on the Cox regression coefficient (*β*) and expression levels of metabolic mRNAs, and the risk scores for each samples were calculated according to the following formula: Σ(ExpmRNAn *×*  *β*mRNAn). Based on the median of risk score, samples were classified into high-risk group and low-risk group. A Kaplan–Meier (K-M) survival curve was plotted to compare the predictive survival time between the two groups. The performance of this signature was evaluated by the area under the curves (AUCs) of the receive operator characteristic (ROC) curve. The risk score was also tested as an independent risk factor with other clinical features by univariate Cox regression and multivariate Cox regression, respectively. During the analyses, the TCGA cohort was used as a training set and the GSE72094 cohort was used as an external testing cohort.

### 2.4. Construction and Validation of a Prognostic Nomogram

A prognostic nomogram including clinical features and risk scores was constructed for TCGA and GEO cohorts, respectively. The calibration plot was graphed to evaluate the prediction probabilities and fitness of the metabolic signature. Finally, a net benefit curve for patients was plotted to reflect the potential utility and evaluate the clinical value of this model by decision curve analysis (DCA).

### 2.5. External Validation of the Prognostic Signature

The genes included in the signature validated their significance in Oncomine database (https://www.oncomine.org/), which provided the meta-analyses of the expression rank for each gene across multiple research. The cBioportal database (https://www.cbioportal.org/) was used to investigate the overview of the alteration that occurred in LUAD for the novel metabolic-related genes. Furthermore, we validated the protein expression levels in the Human Protein Atlas database (https://www.proteinatlas.org/) to compare the differentiation between tumor and control tissues visually.

### 2.6. Gene Set Enrichment Analysis

To investigate the potential molecular mechanism of genes in this signature, we performed the KEGG pathway analysis through Gene Set Enrichment Analysis (GSEA) for the TCGA cohort. The significant pathways were identified with a threshold of FDR <0.05.

### 2.7. Clinical Application in Tumor Microenvironment

We calculated the ESTIMATE scores for each sample and made a comparison between high- and low-risk groups [17]. The ESTIMATE scores contained immune score, stromal score, and tumor purity which, respectively, reflected the infiltration level of immune cells, the stromal content, and the estimated tumor purity. Furthermore, we calculated specific infiltration levels for 22 subtypes of immune cells through the CIBERSORT system to extend the utility of this metabolic signature [18].

### 2.8. Statistical Analysis

All the statistical analyses were conducted by R software (version 3.5.3). The Cox and LASSO regression were employed to screen the survival-related variables. The survival curves were compared by the log-rank test. The differences for the independent samples were analyzed by the Wilcoxon rank-sum test. The coefficient of correlation was calculated by Pearson correlation analysis. *p* < 0.05 was considered statistically significant.

## 3. Results

### 3.1. Identification of Differentially Expressed Metabolic Genes

**Figure 1:**
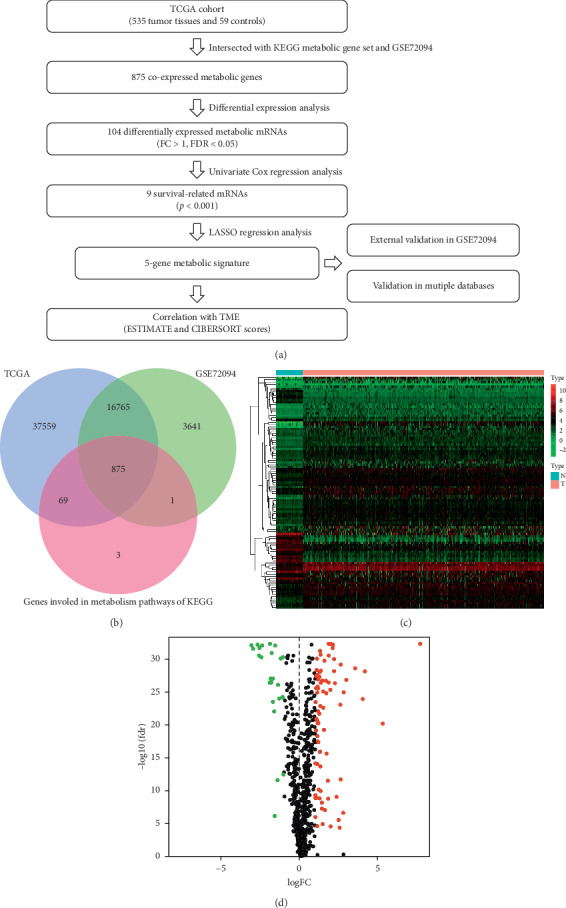
(a) The flowchart for the process of construction and validation to the metabolic gene signature. (b) The intersection for the selected metabolic-related genes. The co-expressed genes among TCGA, GEO, and KEGG metabolism pathway datasets were prepared as metabolic-related genes for the construction of prognostic signature. (c) The heatmap to explain the different expressions between tumor and control groups. (d) The volcano plot of the 104 differentially expressed metabolic mRNAs. Red dots represent 79 upregulated mRNAs, and green dots represent 25 downregulated mRNAs.

We graphed a flowchart to describe our study more visually (Figure 1(a)). A total of 875 metabolic mRNAs were extracted by the intersection of the gene lists from three different databases (Figure 1(b)). Then, 104 differentially expressed metabolic mRNAs (79 upregulated and 25 downregulated) were confirmed between LUAD samples and controls (Figures 1(c) and 1(d)).

### 3.2. Construction and Assessment of the Metabolic Gene Signature

**Figure 2:**
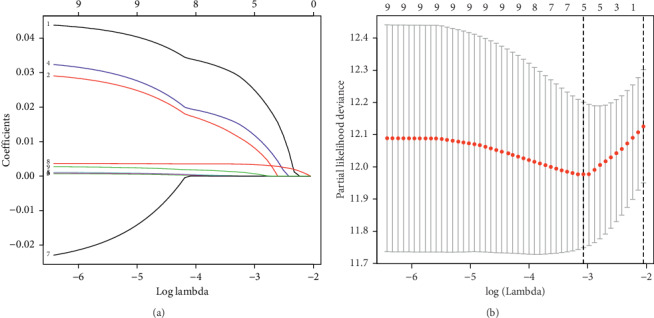
Construction of the metabolic prognostic signature by LASSO regression. (a) The coefficient profiles of 9 prognostic mRNAs. (b) The tuning parameter selection plot of LASSO regression. The dotted lines, respectively, represented the minimum and 1-SE lambda for the optimal volume of variables.

We identified 9 survival-related metabolic mRNAs through the univariate Cox regression analysis. Furthermore, through the LASSO regression, the optimal model was constructed with the least parameters when the lambda was minimum (Figure 2). The five selected genes were as follows: glucosamine 6-phosphate N-acetyltransferase 1 (GNPNAT1), lysophosphatidylglycerol acyltransferase 1 (LPGAT1), thymidylate synthase (TYMS), lactate dehydrogenase A (LDHA), and prostaglandin E synthase (PTGES). The signature was developed by the following formula: Exp (GNPNAT1) × 0.0276 + Exp (LPGAT1) × 0.0102 + Exp (TYMS) × 0.0140 + Exp (LDHA) × 0.0034 + Exp (PTGES) × 0.0010.

**Figure 3:**
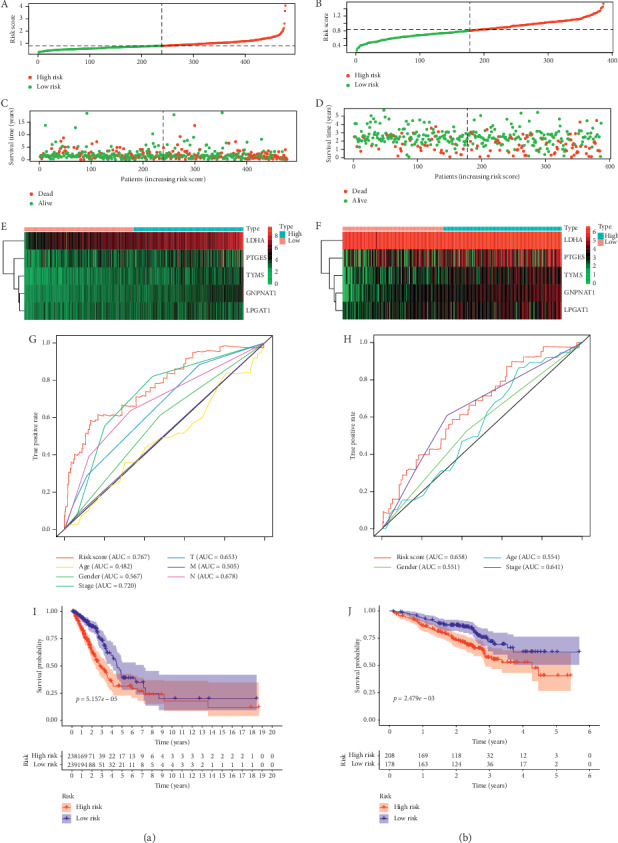
The performance of this signature in training set and testing set. The samples were classified into high- and low-risk group according to the median of risk score in the TCGA cohort. (A, B) The curve of risk score which represented the distribution of patients. (C, D) The dot plot which represented the survival status of patients. (E, F) The heatmap which represented the expression profiling of the 5 genes in the entire dataset. (G, H) The comparison of the AUC to the clinical features and risk scores. (I, J) Kaplan–Meier survival curves for the prediction of prognostic outcomes based on the metabolic gene signature. In both training set and testing set, significant differences were observed in high- and low-risk groups. (a) TCGA cohort. (b) GEO cohort.

**Figure 4:**
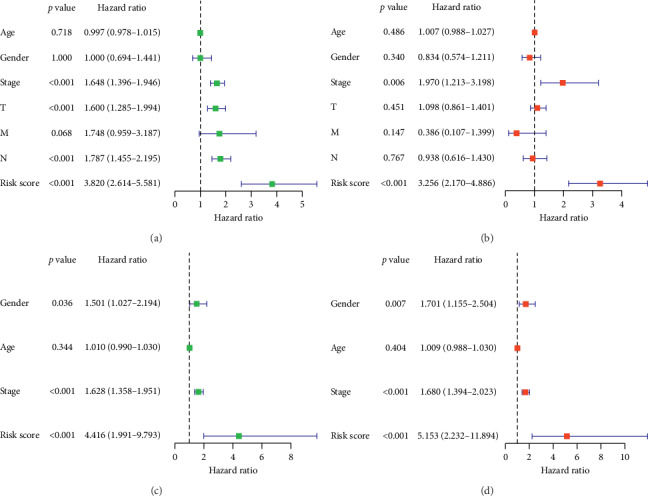
The forest plots of the correlation of clinical features and the prognostic signature with overall survival for LUAD. The univariate Cox regression in the (a) TCGA cohort and (c) GEO cohort. The multivariate Cox regression in the (b) TCGA cohort and (d) GEO cohort.

According to the risk scores, the samples were divided into high-risk group and low-risk group. The AUC of ROC to the risk score was the best in both training set and testing set, and the K-M survival analysis indicated significantly different survival time between the two groups (Figure 3). In both univariate and multivariate Cox regressions, the hazard ratio of risk score was maximal compared with other clinical features. The univariate Cox regression focused on the individual variable but may be affected by the confounding factors. The multivariate Cox regression avoided this limitation. These analyses complemented each other and indicated that the risk score could be a definitely independent risk factor for the prognosis of LUAD (Figure 4).

### 3.3. Construction and Validation of Prognostic Nomograms

**Figure 5:**
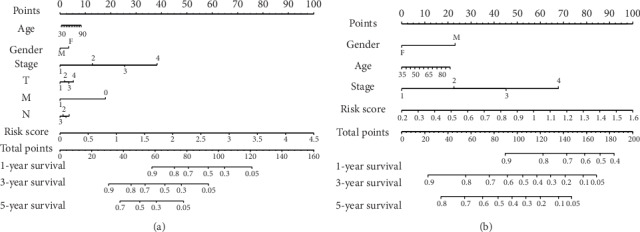
The nomograms for the prediction of overall survival in LUAD. The nomogram consisted of risk score, clinical parameters, and pathological stages. According to this information, a total point for each patient was calculated and it indicated the predictive survival rates. (a) TCGA cohort. (b) GEO cohort.

The nomograms were constructed by the clinical features and risk scores (Figure 5). The C-index was 0.755 (95% confidence interval: 0.706–0.804) and 0.691 (95% confidence interval: 0.636–0.746) for the TCGA and GEO cohorts, respectively. The calibration curves showed the agreement of the models compared with the reference line (Figure S1). The net benefit curves of DCA for the potentiality of clinical application confirmed that the model could provide satisfactory benefits (Figure S2). Generally speaking, our model had an approximately moderate accuracy in both TCGA and GEO cohorts and might increase the sensitivity and specificity in the prognostic prediction of LUAD to some extent.

### 3.4. External Validation of the Prognostic Signature

**Figure 6:**
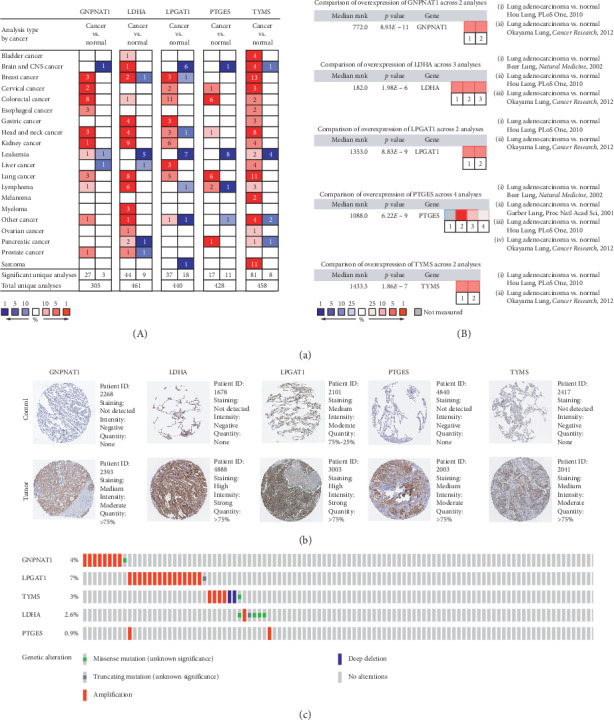
Validation among multiple databases from different levels. (a) The expression profiling of the five genes in Oncomine database. (A) The expression levels among different cancers. (B) The expression levels of the five genes based on the published research. The *p* value for a gene is derived from the median-ranked analysis. The rank for a gene is the median rank for that gene across each of the analyses. (b) The comparison of protein expression of the five genes in control and tumor tissues in the Human Protein Atlas database. (c) The alterations of the five genes based on the cBioportal database.

All of the five genes were confirmed the significantly different expression in Oncomine database (Figure 6(a)), which was consistent with our results. With respect to protein levels, they were also significantly differentially expressed between LUAD and control tissues (Figure 6(b)). In the Gene Alteration Atlas, LPGAT1 possessed the most occurrence of mutation with 7% among the samples and other genes also showed alterations; it might clarify the aberrant expression between LUAD and control samples (Figure 6(c)). The correlation between LUAD and the five genes was further confirmed through the validation among multiple databases.

### 3.5. Gene Set Enrichment Analysis

**Figure 7:**
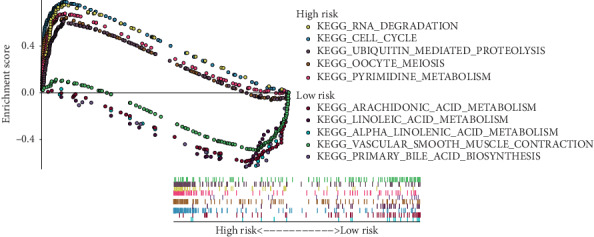
The top 5 significant enriched KEGG pathways in the TCGA cohort.

We performed the GSEA and identified the enriched KEGG pathways (Figure 7). With respect to the high-risk group, the pathways were mainly associated with cell proliferation and the top 5 pathways were RNA degradation, cell cycle, ubiquitin-mediated proteolysis, oocyte meiosis, and pyrimidine metabolism. With respect to the low-risk group, the pathways were mainly associated with the lipid metabolism and the top 5 were arachidonic acid metabolism, linoleic acid metabolism, alpha linolenic acid metabolism, vascular smooth muscle contraction, and primary bile acid biosynthesis. The results revealed different metabolic features in the risk groups.

### 3.6. Correlation with Tumor Microenvironment

**Figure 8:**
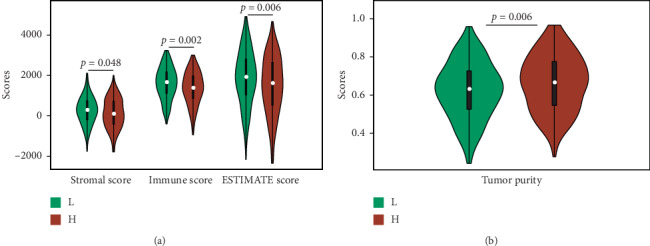
The comparison of ESTIMATE scores. The green part represented the low-risk group, and the red part represented the high-risk group. (a) The infiltration level of the immune cells, the stromal content, and the total scores. (b) The estimated tumor purity.

**Figure 9:**
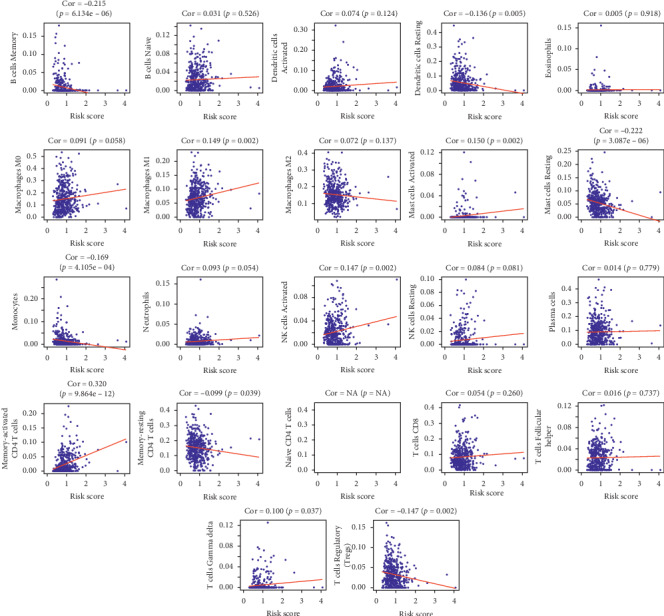
The relationships between the prognostic signature and the infiltration levels of 22 subtypes of immune cells calculated by the CIBERSORT system. The infiltration level of CD4+ naive T cell in LUAD samples was not calculated in our study, and the correlation coefficient was NA.

The samples in the low-risk group possessed a higher stromal score, immune score, and total score compared with those of the high-risk group; meanwhile, the tumor purity also had a significant difference between the two groups (Figure 8). Through the CIBERSORT system, we calculated the relationship between this prognostic signature and the infiltration levels of immune cells (Figure 9). A total of 11 subtypes of immune cells (memory B cells, resting dendritic cells, macrophages M1, activated mast cells, resting mast cells, monocytes, activated NK cells, memory-activated CD4 T cells, memory-resting CD4 T cells, gamma delta T cells, and regulatory T cells) had obvious relationships between the infiltration levels and risk scores.

## 4. Discussion

Although great achievements about new therapeutic strategies have been reported in the past decades, the overall survival rates of LUAD remains unsatisfactory [1, 19].The pathological subtypes presented limitations in the prediction of prognosis. Patients could have totally different final outcomes although they might possess similar clinical and pathological types [20]. The development of next-generation sequencing promoted the preclinical application of bioinformatics [21, 22], which could comprehensively combine the gene profiling with the clinical parameters. Compared with the tumor-node-metastasis (TNM) system, it has been confirmed among various types of cancers that the prognostic signature could improve the accuracy of prediction [23–25].

In the field of LUAD, there were several previous studies which had successfully constructed the prognostic signature from different perspectives [26–28]. Compared with these studies, we extracted the related genes and developed a metabolic prognostic signature. We, respectively, confirmed its accuracy and robustness in the training set and testing set. Our signature could efficiently identify the overall survival time for different risk groups and was further validated from different levels in multiple databases. Moreover, we explored the correlation between the signature and TME in order to expand the clinical application and provide more clues for the choice of therapeutic strategy.

Previous research about NSCLC has reported the biological function and expression patterns for the model genes. GNPNAT1, also known as GNA1, was a key member involved in the biosynthesis about acetylglucosamine and it was confirmed that the underexpression of GNPNAT1 could result in the inhibition of infiltration and adhesion of lung cancer cells [29]. LDHA was an important enzyme that participated in the cell energy metabolism which promoted the malignant behavior and predicted poor survival outcomes in LUAD [30, 31]. PTGES was an enzyme that was mostly involved in the inflammation response. It was also reported that the aberrant expression in the NSCLC cell lines and PTGES knockdown could significantly suppress the migration of lung cancer cell [32, 33]. TYMS played an essential role during the DNA synthesis, the alteration of TYMS might increase the risk of lung cancer, and the expression of TYMS confirmed the correlation with EGFR mutation in LUAD patients [34, 35]. LPGAT1 was reported as a novel gene that mainly participated in the lipid metabolism and confirmed the role in influencing BMI and body fat [36]. Previous researches revealed that LPGAT1 was differentially expressed between normal and tumor tissue and might be potential targets for crucial microRNAs in LUAD [37, 38]. Our research indicated that LPGAT1 was a metabolic-related gene with a potential prognostic value, and it might be a novel diagnostic and therapeutic target for LUAD. Significant alteration of amplification in LPGAT1 was observed, and different protein expression levels were also confirmed between the tumor tissue and control.

The difference of enriched pathways between high- and low-risk groups was revealed by GSEA. The results indicated that the two risk groups possessed significantly different metabolic features. Pathways in the high-risk group were mainly correlated with the cell proliferation, and in the low-risk group, they were correlated with lipid metabolism. Through the differences with them, we could partly acquire the different metabolic features identified by risk scores and the underlying molecular mechanisms. It could be a cost-effective complementary tool that indicates the metabolic microenvironment and the prognosis.

Besides the differences in metabolic features, the prognostic signature could also reveal corresponding changes in the TME, which was showed as ESTIMATE scores and CIBERSORT scores. Within the TME, along with the tumorigenesis and progression, the aberrant of tumor metabolism could implicate the immunosuppression and tumor cells could escape from the immune response [39]. Through the reflection to the composition of TME by this signature, we could conveniently monitor the infiltration of immune cells and further reduce the degree of immune response. This signature could reflect these changes of TME from different aspects and had the potentiality to be an appropriate assistance for rational diagnosis and individualized treatment. Meanwhile, it was confirmed that the therapies which were targeted on the tumor metabolism and tumor immune check point in TME had certain antitumor effects [40]. It could be a new prospective direction for the challenge of drug-resistance and might provide complementary clues during the application of immunotherapy.

The limitations of the current TNM system were gradually recognized in recent years. To improve the clinical application, a new method of immunoscore system was recommended by the international consensus in the field of colon cancer [41]. This immunoscore was derived from the density of CD3+ and CD8+ T-cell effectors and was validated to be satisfactory performance in the prediction of recurrence and prognosis. It was considered as a complementary risk factor alongside the TNM system for the classification of tumor and was called the TNM-I system. In the field of NSCLC, the similar research was in progress and had got preliminary achievements [42]. We calculated the relationship between our signature and the infiltration levels of 22 subtypes of immune cells in TME, and half of them were significantly correlated. Through this signature, we could correlate the distribution of immune cells with the prognosis of patients. Our signature provided innovative perspectives from the angle of tumor metabolism. The comprehensive reflection of the TME, especially the correlation between this prognostic signature and the infiltration of immune cells, could enrich the research on the TNM-I system, and the role in prognostic prediction could provide more details for the research of this new immunoscore system.

There were still several limitations to our research. First, it was difficult to reflect the whole landscape of the tumor metabolism based on the transcriptomics data, and our analyses were just focused on the particular aspect. Second, although our signature had been validated among multiple databases, the experimental exploration was still needed to further confirm the accuracy and clinical utility. Third, the composition of cohort from TCGA and GEO database were mainly white and black; the extension to other races is still needed to be validated.

## 5. Conclusions

In conclusion, we successfully developed a robust metabolic-related gene signature for the prognostic prediction of LUAD based on the TCGA and GEO database. Our signature could reflect the metabolic features and the status of TME for LUAD.

## Data Availability

The datasets used and/or analyzed during the current study are available from the corresponding author upon reasonable request.

## Abbreviations

ALK: Anaplastic lymphoma kinase
AUC: Area under the curves
DCA: Decision curve analysis
EGFR: Epidermal growth factor receptor
FC: Fold change
FDR: False discovery rate
GEO: Gene Expression Omnibus
GNPNAT1: Glucosamine 6-phosphate N-acetyltransferase 1
GSEA: Gene set enrichment analysis
KEGG: Kyoto Encyclopedia of Genes and Genomes
K-M: Kaplan–Meier
LASSO: Least absolute shrinkage and selection operator
LDHA: Lactate dehydrogenase A
LPGAT1: Lysophosphatidylglycerol acyltransferase 1
LUAD: Lung adenocarcinoma
NSCLC: Non-small-cell lung cancer
PTGES: Prostaglandin E synthase
ROC: Receiver operator characteristic
TCGA: The Cancer Genome Atlas
TME: Tumor microenvironment
TYMS: Thymidylate synthase.

## Supplementary Materials

Supplementary Materials Figure S1: the calibration curves of nomograms. The *X* axis represents the predicted survival probability and the *Y* axis represents the actual survival probability. (A) 3-year calibration curve of the TCGA cohort. (B) 5-year calibration curve of the TCGA cohort. (C) 3-year calibration curve of the GEO cohort. (D) 5-year calibration curve of the GEO cohort. Figure S2: The DCA curves of nomograms. When the value of the dotted curve was more than that of the gray curve, the application of this model adds benefits for the patients. (A) 3-year DCA curve of the TCGA cohort. (B) 5-year DCA curve of the TCGA cohort. (C) 3-year DCA curve of the GEO cohort. (D) 5-year DCA curve of the GEO cohort.
