# Supplementary material for: Identification of a 5-Gene Metabolic Signature for Predicting Prognosis Based on an Integrated Analysis of Tumor Microenvironment in Lung Adenocarcinoma
Source: J Oncol. 2020 Jun 26;2020:5310793. doi: 10.1155/2020/5310793 (PMC7335383; doi:10.1155/2020/5310793)
Supplement: Supplementary Materials — Figure S1: the calibration curves of nomograms. The X axis represents the predicted survival probability and the Y axis represents the actual survival probability. (A) 3-year calibration curve of the TCGA cohort. (B) 5-year calibration curve of the TCGA cohort. (C) 3-year calibration curve of the GEO cohort. (D) 5-year calibration curve of the GEO cohort. Figure S2: The DCA curves of nomograms. When the value of the dotted curve was more than that of the gray curve, the application of this model adds benefits for the patients. (A) 3-year DCA curve of the TCGA cohort. (B) 5-year DCA curve of the TCGA cohort. (C) 3-year DCA curve of the GEO cohort. (D) 5-year DCA curve of the GEO cohort. [file 5310793.f1.docx]

## Supplementary Materials


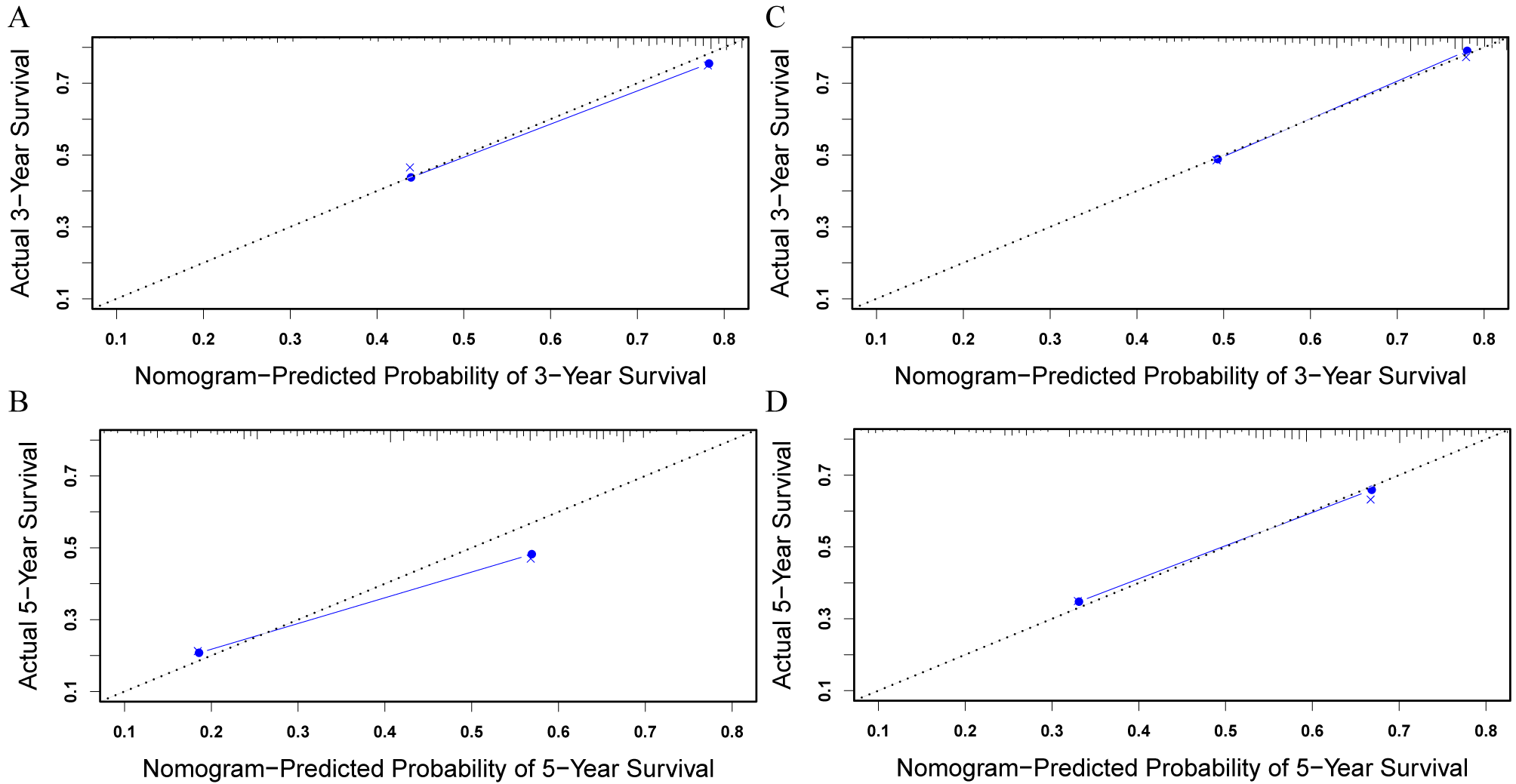


Figure S1 The calibration curves of nomograms. The X axis represented the predicted survival probability and the Y axis represented the actual survival probability. (A) 3-year calibration curve of TCGA cohort. (B) 5-year calibration curve of TCGA cohort. (C) 3-year calibration curve of GEO cohort. (D) 5-year calibration curve of GEO cohort.


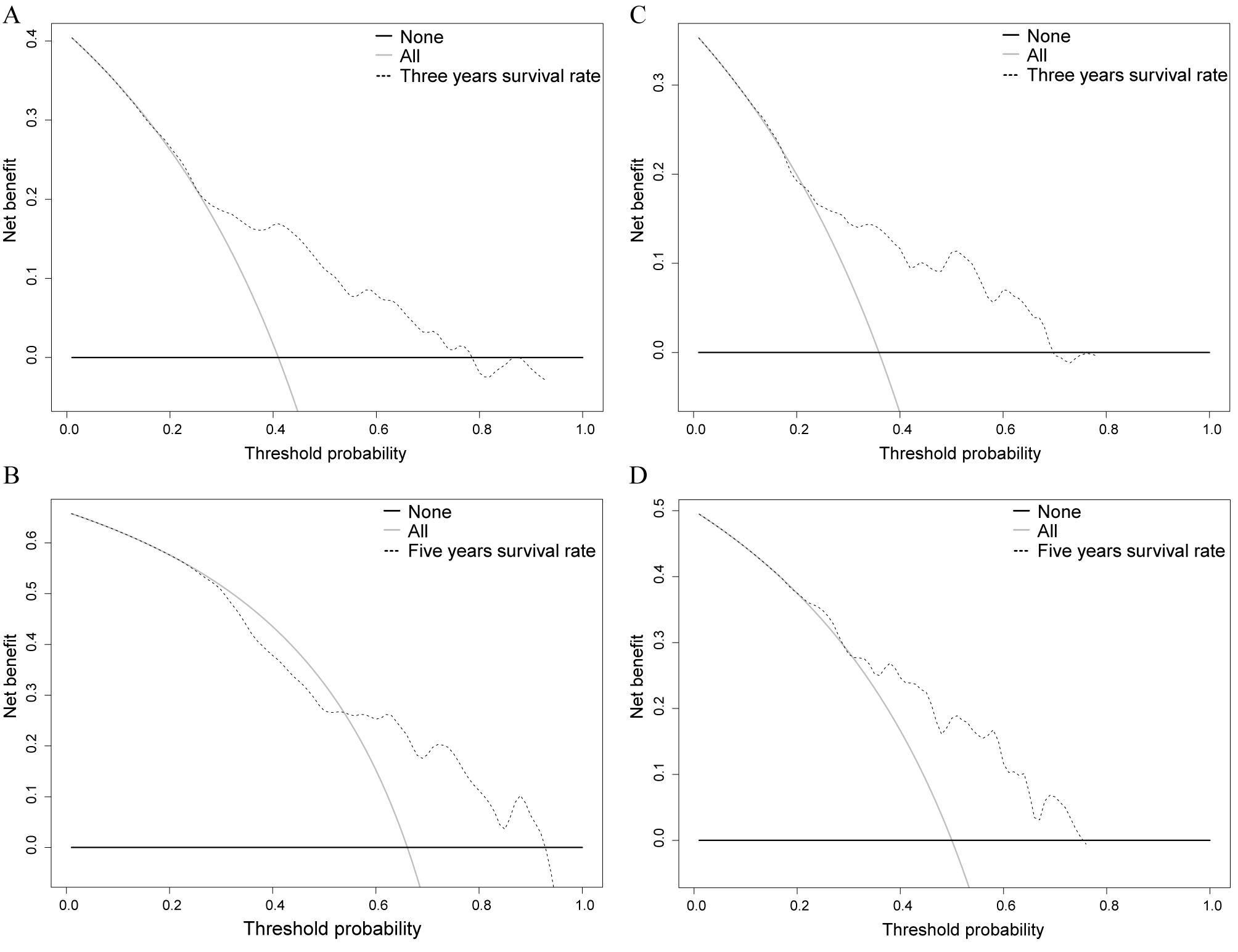


Figure S2 The DCA curves of nomograms. When value of the dotted curve was more than gray curve, the application of this model could add benefits for patients. (A) 3-year DCA curve of TCGA cohort. (B) 5-year DCA curve of TCGA cohort. (C) 3-year DCA curve of GEO cohort. (D) 5-year DCA curve of GEO cohort.
